# Rupturing Giant Plasma Membrane Vesicles to Form Micron-sized Supported Cell Plasma Membranes with Native Transmembrane Proteins

**DOI:** 10.1038/s41598-017-15103-3

**Published:** 2017-11-09

**Authors:** Po-Chieh Chiang, Kevin Tanady, Ling-Ting Huang, Ling Chao

**Affiliations:** Department of Chemical Engineering, National Taiwan University, Taipei, Taiwan

## Abstract

Being able to directly obtain micron-sized cell blebs, giant plasma membrane vesicles (GPMVs), with native membrane proteins and deposit them on a planar support to form supported plasma membranes could allow the membrane proteins to be studied by various surface analytical tools in native-like bilayer environments. However, GPMVs do not easily rupture on conventional supports because of their high protein and cholesterol contents. Here, we demonstrate the possibility of using compression generated by the air-water interface to efficiently rupture GPMVs to form micron-sized supported membranes with native plasma membrane proteins. We demonstrated that not only lipid but also a native transmembrane protein in HeLa cells, Aquaporin 3 (AQP3), is mobile in the supported membrane platform. This convenient method for generating micron-sized supported membrane patches with mobile native transmembrane proteins could not only facilitate the study of membrane proteins by surface analytical tools, but could also enable us to use native membrane proteins for bio-sensing applications.

## Introduction

Previous studies have derived giant plasma membrane vesicles (GPMVs)^1–5^ from cells in order to study membrane protein behaviors and lipid raft behavior^6,7^. GPMVs are blebs taken directly from the cell plasma membrane that contain lipid bilayers and the embedded membrane proteins^8^. They do not contain the other biological components of the cell and can be used as a model system to study any cell plasma membrane-related behaviors of interest. However, GPMVs have spherical configurations and easily rotate and move in solutions. Thus, it would be desirable to deposit the GPMVs to form planar supported membranes. The planar geometry of the platform is compatible with a wide range of surface analytical tools requiring planar geometry. More importantly, the connected and fluid supported membranes could provide the possibility of collecting transmembrane proteins from numerous GPMVs and allowing them to be freely transported in the bilayer platform for protein characterization purposes and for various sensing applications^9,10^.

One challenge in using native membrane vesicles^11–14^ to form a supported lipid bilayer (SLB) is that they do not easily rupture on conventional supports because of their high protein and cholesterol contents^15,16^. Some methods have used microfluidics to create a high energy edge for the lipid bilayer^15^, added viral fusion peptides^17^, and mixed the samples with easily ruptured synthetic lipid vesicles^6,8^ in order to facilitate the rupture of native membrane vesicles. These methods use membrane vesicles with sizes of tens to hundreds of nanometers because small vesicles have high curvature which can facilitate the vesicle rupture^7,18^. However, even when the membrane vesicles have high curvature, all of these methods still require the addition of synthetic lipid vesicles to facilitate the vesicle rupture. The process can dilute the components in the plasma membrane, and it is difficult to measure the actual deposition amount of plasma membranes on a support. Several other approaches, such as the freeze-thaw process^19^ and solvent-exchange deposition^20,21^, have also been developed to successfully induce adsorbed lipid vesicles with unfavorable lipid compositions to rupture to form SLBs. However, these methods have not been demonstrated to rupture lipid vesicles with membrane proteins, and the freeze-thaw step and additional solvent could influence membrane protein structure and function.

Herein, we developed a method involving the use of air-water interfaces to compress and rupture GPMVs to form cell-sized supported plasma membranes, which size is suitable for the events occurring in the plasma membrane to be microscopically observed and characterized. The GPMV patch coverage can be significantly increased by applying multiple air-water interface treatments. The GPMV patch generated after our air-water interface treatment has similar fluorescence intensities and membrane diffusivities as a spontaneously ruptured GPMV patch, suggesting that the membrane integrity can remain after the treatment. In addition, we used the immunostaining of Aquaporin 3 (AQP3), a functional transmembrane protein which allows water molecules to be transported through the plasma membranes of cells^2,22–25^, to demonstrate that the native transmembrane proteins in HeLa cells can be incorporated into and remain mobile in the supported plasma membrane patch.

## Results and Discussions

### Use of the GPMVs from HeLa cells to form planar supported plasma membrane patches

**Figure 1:**
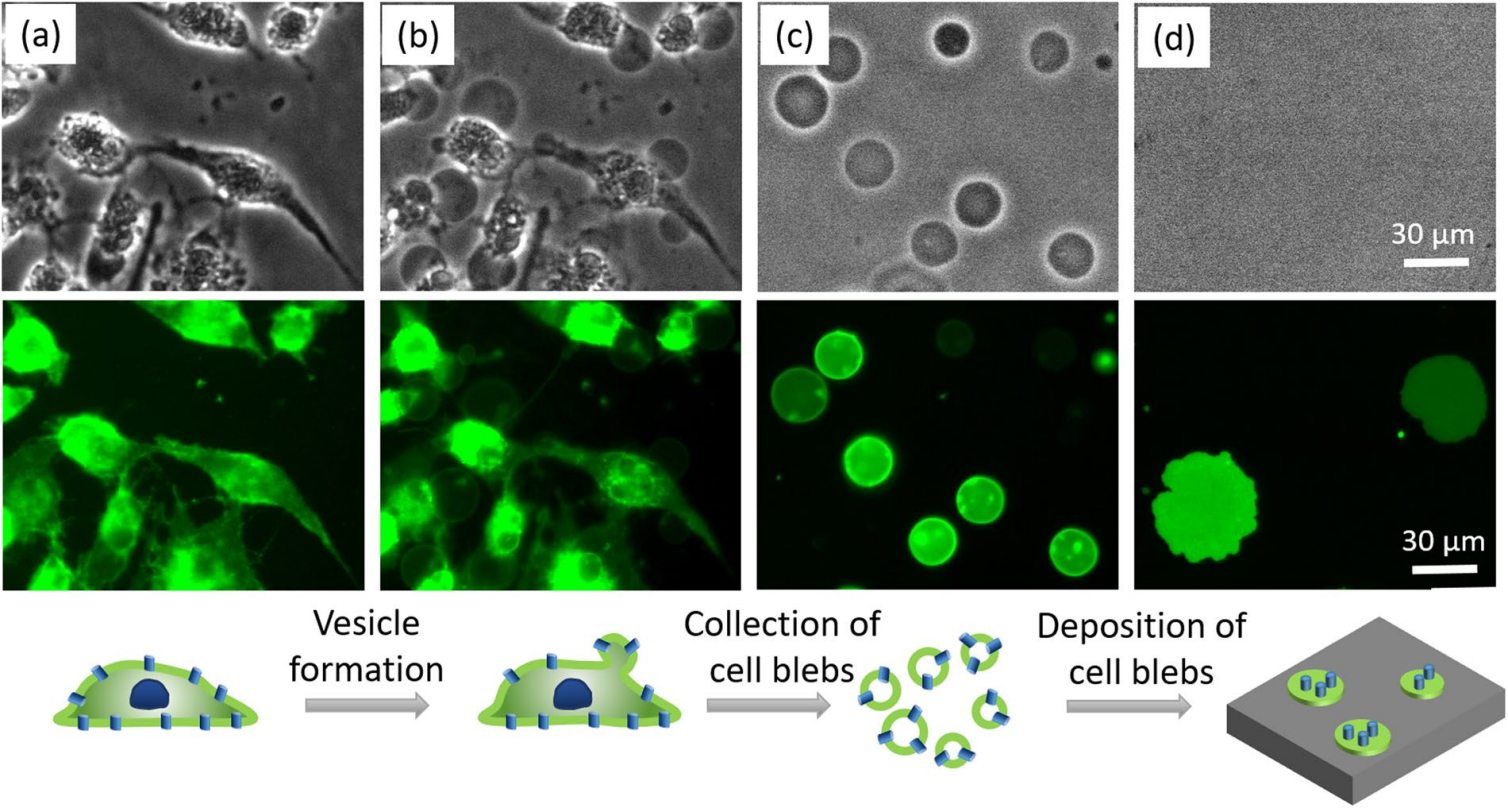
Obtaining giant plasma membrane vesicles (GPMVs) from cells to form supported plasma membrane patches. (**a**,**b**) HeLa cell culture before and 3 hr after the addition of vesiculation reagents; (**c**) collection of GPMVs; (**d**) planar plasma membrane patches on a glass support. Top panel: bright field images; middle panel: fluorescent images revealed by Fast DiO; bottom panel: illustration of the process.

We used a chemical vesiculation method^2,26^ to obtain giant plasma membrane vesicles (GPMVs) from HeLa cells, and deposited the collected GPMVs on a glass support. Figure 1 shows the bright field images (top panel), the fluorescence images (middle panel), and an illustration (bottom panel) of the process. Figure 1(a) shows the cells before the addition of the vesiculation reagent and Fig. 1(b) shows that micron-sized GPMVs blebbed out from the cells. The blebbed GPMVs were collected (Fig. 1(c)) and added to a clean glass coverslip for deposition. After incubating the GPMVs with a clean glass coverslip for 1.5 hr in a well covered with a lid to prevent the water evaporation, we observed that only a few GPMVs can spontaneously rupture to form a planar patch on the glass support (Fig. 1(d)). We labeled the HeLa cell membrane with Fast-DiO since the ruptured GPMV patches cannot be clearly observed by bright field microscopy. The fluorescence intensity variation among the GPMVs and the ruptured patches was probably due to the different cellular uptake amounts of Fast-DiO.

### GPMVs ruptured by an air-water interface treatment

**Figure 2:**
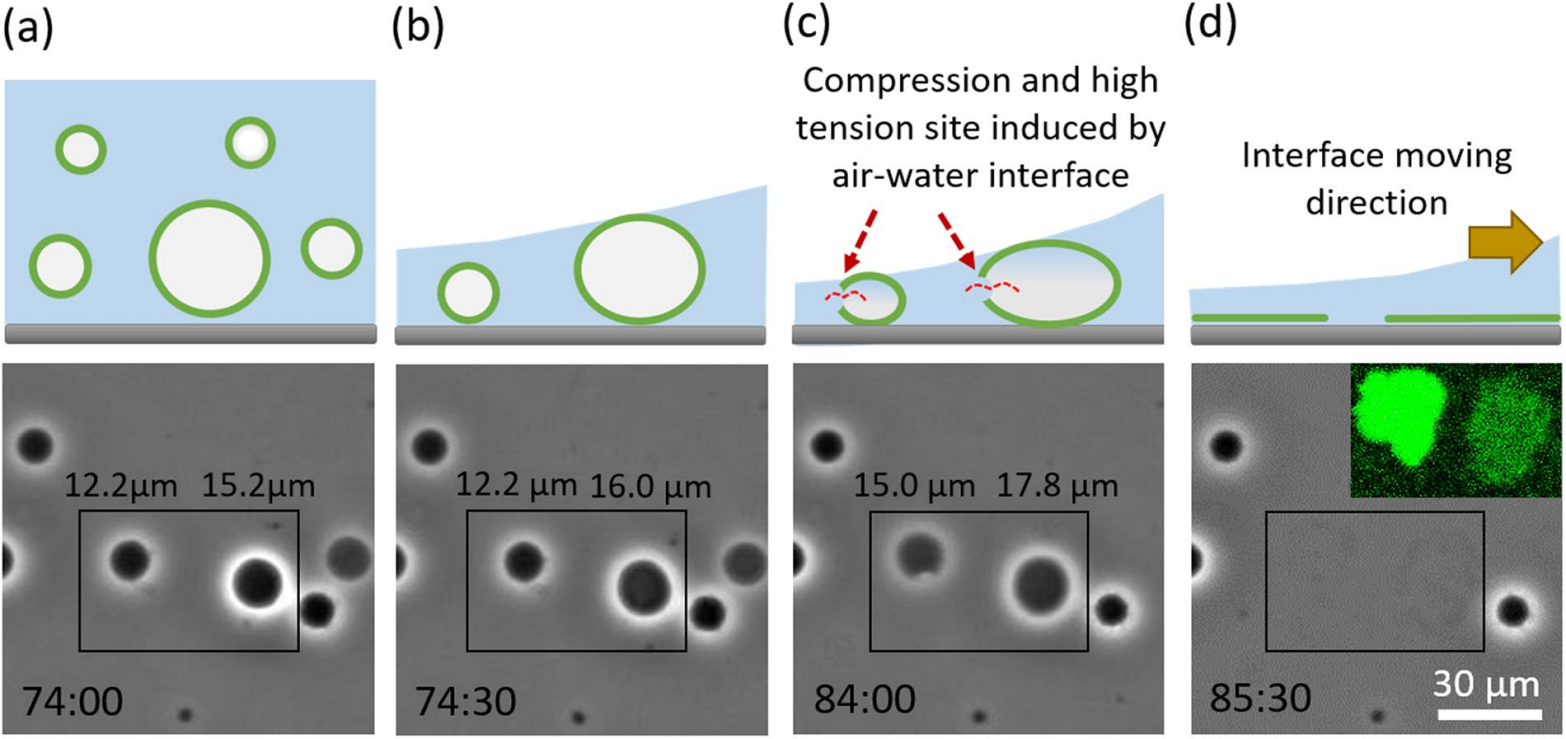
GPMVs ruptured by an air-water interface treatment when the interface approached from the left side and continued moving to the right side of the image. (**a**) Before the GPMVs were influenced by the air-water interface; (**b**) the GPMV on the right in the box was compressed by the air-water interface; (**c**) the GPMV on the left was also compressed; (**d**) the GPMVs had ruptured to form planar supported membrane patches. The inset image is the fluorescence image taken at the location of the box.

We observed that the ruptured GPMV patches can be significantly increased if we allow the solution in the well to evaporate and generate an air-water interface passing along the glass surface. Figure 2 shows how the GPMVs responded when an air-water interface approached from the left side and continued moving to the right side of the image (see Supplementary video). Before the air-water interface approached, the GPMVs close to the glass coverslip looked spherical and could be clearly distinguished under a phase contrast microscope. When the air-water interface approached, we observed that the projected area of some of the GPMVs increased and then ruptured, suggesting that they were compressed by the air-water interface.

Figure 2(a) shows the image of typical GPMVs before any compression occurred. The projected area diameter of the GPMV on the left in the box is around 12.2 μm and the one on the right is around 15.2 μm. At the time point of Fig. 2(b), the projected area diameter of the GPMV on the right side became 16.0 μm while the one on the left remained the same, suggesting that the air-water interface already reached the top of the larger GPMV on the right and started to compress the GPMV. At the time point of Fig. 2(c), the smaller GPMV on the left was also compressed and the projected area diameter became 15.0 μm, while the larger one was further compressed and the diameter became 17.8 μm. In addition, a defect or pore, which could be a rupture site induced by the high curvature due to the vesicle compression, was observed in the GPMV on the left. At the time point of Fig. 2(d), the GPMVs had ruptured to form planar supported membrane patches and therefore cannot be clearly observed in the bright field image. The inset fluorescence image taken at the location of the box shows that two ruptured patches had indeed formed in that location.

A previous study by Hamai *et al*.^27^ has demonstrated that the spontaneous rupture of a giant unilamellar vesicle (GUV) can be induced by the vesicle deformation due to the favourable surface adsorption energy. The high bending curvature caused by the deformation can increase the possibility of pore formation and then initiate the rupture process. From their images, we estimated the vesicle sizes and calculated that the deformation extent (the ratio of the projected area diameter of the deformed vesicle right before the rupture to the one before the deformation) is around 1.22. We analysed our image data and found that the average deformation extent of those GPMVs rupturing when the air-water interface passed through is 1.21 ± 0.03 (see Supplementary Fig. S1 and Table S1). The similar deformation extent suggests that the rupture follows a similar mechanism. Different from the conventional spontaneous rupture of a GUV as shown in the previous study^27^, a GPMV is usually more rigid because of the high protein content and the surface adsorption energy between the GPMV and the glass support may be not strong enough for the GPMV to spontaneously deform to the extent for the pore formation. The air-water interface could be used to further compress GPMVs to increase the deformation extent and therefore initiate the rupture process.

One thing to note is that we used an evaporation method to create the air-water interface, and a high salt concentration in the solution can occur after most of the water has evaporated. To eliminate the possibility that the hypotonic osmolality pressure applied to the GPMVs caused by the evaporation was the reason for the increased coverage, we added a saturated sodium chloride solution to a well with GPMVs to create a hypotonic osmolality pressure without generating an air-water interface and found no significant increase in the coverage ratio compared with that yielded by the incubation method. The osmolarity shock is not that useful probably because the GPMV membranes contain ion channels and therefore could quickly balance the osmolarity inside and outside the GPMVs.

### Membrane coverage increased by applying multiple air-water interface treatments

**Figure 3:**
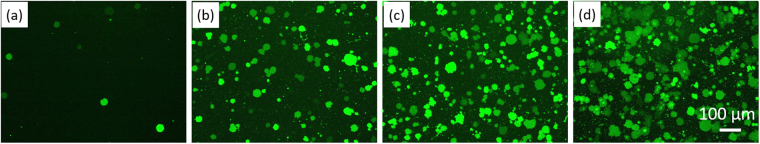
Coverage ratio of GPMV patches on the support caused by different methods. (**a**) Incubating for 1.5 hr to allow the GPMVs to spontaneously rupture; (**b**–**d**) applying the air-water interface treatment once, twice, and three times, respectively.

Figure 3 shows the membrane coverage if the GPMVs were allowed to spontaneously rupture versus the membrane coverage if the air-water interface treatment was applied from one to three times. The membrane coverage was low (1.1 ± 0.3%) when the GPMVs were incubated with the support for 1.5 hr to allow for their spontaneous rupture. Figure 3(b) shows that the membrane coverage was significantly increased to 11 ± 1% after a single application of the air-water interface treatment. The comparison of Fig. 3(a) and (b) shows that the air-water interface helped increase the overall rate of rupture. In addition, Fig. 3(c) and (d) show that the membrane coverage was further increased when we loaded a fixed amount of GPMV solution for each air-water interface treatment and applied the treatment twice (25 ± 4%) and three times (35 ± 5%), respectively. These results show that multiple air-water interface treatments can be used to significantly increase the coverage ratio.

### The membrane integrity of GPMV patches after an air- water interface treatment

Two important questions regarding the use of an air-water interface to rupture the GPMVs is whether the interfacial force would peel off the membrane and how stable the bilayer is after the air-water interface treatment. To examine whether the bilayer structure of the GPMV patch remained after our air-water interface treatment, we compared the lipid membrane diffusivity and the fluorescence intensity of the spontaneously ruptured GPMV patches with those of the GPMV patches ruptured by the air-water interface. Note that each GPMV already has different fluorescence intensity before the air-water interface treatment probably because of the different cellular uptake amounts of Fast-DiO, and therefore we used the averaged intensity of 70 patches to represent the membrane amount of a sample and observed how the averaged fluorescence intensity is influenced by the air-water interface treatment.

**Figure 4:**
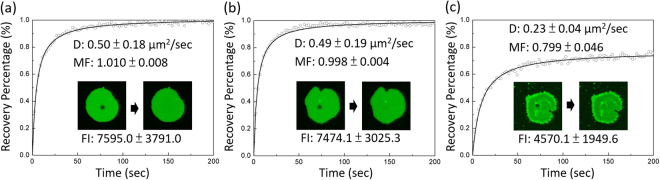
FRAP measurement and fluorescence intensity measurement to examine the integrity of (**a**) a spontaneously ruptured GPMV patch, (**b**) a GPMV patch ruptured by the air-water interface and rehydrated right after the air-water interface passed through the entire sample, and (**c**) a GPMV patch ruptured by the air-water interface and rehydrated after 1.5 hr. D: membrane diffusivity by Fast-DiO. MF: mobile fraction. FL: fluorescence intensity. Data are represented as means with standard deviation (n = 9 from three samples (three patches per sample) for D and MF; n > 70 from more than three samples for FI).

Figure 4(a) and (b) show that the averaged fluorescence intensity and averaged lipid membrane diffusivity of the sample consisting of GPMVs broken by the air-water interface were similar to those of a sample consisting of spontaneously ruptured GPMV patches, suggesting that the lipid membrane structures of the GPMV patches were similar and probably not destroyed by our air-water interface treatment. The reason that the air-water interface did not ruin our membranes was probably because a GPMV membrane has many membrane proteins and can thus maintain some water and because the abundant proteins might also increase the rigidity of the membrane to prevent it from being peeled off by the air-water interfacial tension. Note that the lipid diffusivity in the supported GPMV patch reported here is around one order of magnitude smaller than the previously reported diffusivity in a free-standing GPMV^28^. Similar decrease in diffusivity has been also reported when a giant unilamellar vesicle (GUV) was deposited onto a support^29,30^.

Note that although the rigidity of the membrane may prevent it from being peeled off by the air-water interfacial tension, we found that the bilayer structure may not be stable after the sample is exposed to air for a long time. In a typical treatment, the sample started to dry from the middle region of the sample and the earliest dried middle region was exposed to air for about 1 hr and all of the other regions were exposed to air for less than 1 hr. Our result in Fig. 4(b) shows that the GPMV patch in the middle region still had similar diffusivity and intensity after the rehydration. However, if the GPMV patch was rehydrated after the region was exposed to air for 2.5 hr, the intensity, diffusivity and mobile fraction significantly decreased, as shown in Fig. 4(c). The comparison of Fig. 4(b) and (c) suggests that the GPMV patch bilayer can remain stable in air for an hour but the structure may significantly change if the sample is exposed to air for longer time.

### Mobile native transmembrane protein, Aquaporin 3, in the supported plasma membrane

**Figure 5:**
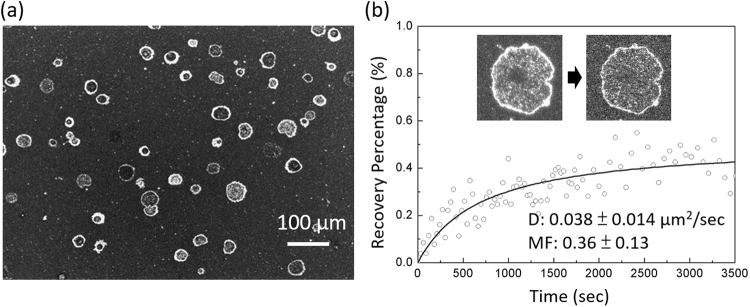
(**a**) The experimental fluorescence images of anti-AQP3. (**b**) FRAP data measurement to obtain anti-AQP3 diffusivity in the GPMV patch region with the surrounding region blocked by BSA. D: regressed diffusivity. MF: mobile fraction. Data are represented as means with standard deviation (n = 9 from three samples (three patches per sample)).

To further confirm that the GPMV patches do contain native membrane proteins, we used immunostaining to label an abundant membrane protein in HeLa cell plasma membrane, Aquaporin 3 (AQP3). The fluorescence image of Anti-AQP3 in Fig. 5(a) suggests that abundant AQP3 exist in our supported membrane patches. Our control experiment shows that the nonspecific binding of the antibody to the lipid membrane is weak (see Supplementary Fig. S3) and we did not use any fluorescence dyes or probes in the samples to measure the AQP3 diffusivity, eliminating the possibility that the measured diffusivity is due to the non-specific binding of anti-AQP3 to mobile lipids or due to the bleed-through in fluorescence imaging from a mobile lipid probe. The FRAP data in Fig. 5(b) show that the diffusivity of AQP3 measured in the GPMV patch is 0.038 ± 0.0014 μm^2^/sec. The diffusivity of the membrane protein is one order of magnitude smaller than the diffusivity of the lipid dye (0.49 ± 0.195 μm^2^/sec as shown in Fig. 4(b)), which is consistent with a previously reported FRAP-measured diffusivity of an over expressed transmembrane protein, β-secretase 1 (BACE1) in a supported membrane^6^. The significant immobile fraction of membrane proteins is probably due to the interaction of the extruding hydrophilic domain of AQP3 with the glass support^31^.

The AQP3 antibody we bought binds to the cytosolic domain of AQP3^32^. Therefore, the binding situation suggests that most of the ruptured GPMV patches have cytosolic side facing up. We also noticed that many antibodies bound to the boundaries of the GPMV patches. The FRAP results show that these antibodies were mobile (see Supplementary Fig. S4), indicating that the bright rims were not just due to the non-specific binding of antibodies to the patch boundaries and many AQP3s located at the patch boundaries in these systems. Membrane proteins are soft entities, and previous studies have shown that proteins can change their conformation and spread their hydrophobic amino acid residues over the hydrophobic surface in an aqueous environment to reduce the net hydrophobic surface area of the system exposed to water^33–35^. Therefore, we hypothesized that the trapping of AQP3 at the membrane patch boundaries to protect the exposed lipid hydrophobic acyl chains may decrease the entire system energy and is a thermodynamically favourable process.

Note that although previous two studies^6,8^ can already form supported membranes with membrane vesicles from cells, they used submicron-sized vesicles and required the addition of synthetic lipid vesicles to facilitate the bleb rupture. With their methods, the supported membranes are diluted with synthetic lipids and it is challenging to quantify and further increase the actual deposition amount of the cell membranes. In this study, we used air-water interface to rupture cell-sized GPMVs and therefore do not need to dilute the plasma membrane components with synthetic lipids in a supported membrane. In addition, a single plasma membrane patch with a size of tens of microns could facilitate the events occurring in the membrane to be microscopically observed and facilitate the generation of pore-spanning membranes over structured supports for various applications^36–41^.

## Materials and Methods

### Cell culture and labeling

HeLa cells were cultured at 37 °C and 5% CO_2_ in Dulbecco’s modified eagle medium (DMEM, HyClone, USA) containing 10% fetal bovine serum (FBS, Biological Industries, USA), 100 units/ml antibiotic antimycostic solution (Sigma-Aldrich, USA), 1 mM sodium pyruvate (HyClone, USA), and 1.5 g/L sodium bicarbonate (HyClone, USA). Prior to the GPMV formation step, cell membranes were stained with 5 μg∕mL of ClO_4_ (3,3′-Dilinoleyloxacarbocyanine Perchlorate) (Fast-DiO, Thermo Fisher Scientific, USA) for 10 min.

### GPMV formation and collection

GPMVs were prepared using a previously reported protocol^2,26^. In brief, after the cell labeling, the cells were washed three times with phosphate buffered saline (PBS) consisting of 137 mM NaCl, 2.7 mM KCl, 10 mM Na_2_HPO_4_, and 2 mM NaH_2_PO_4_ at pH 7.4, followed by being washed twice with GPMV buffer (2 mM CaCl_2_/10 mM Hepes/150 mM NaCl, pH 7.4). Later, the cells were incubated with GPMV vesiculation buffer (25 mM paraformaldehyde (PFA) and 2 mM dithiothreitol (DTT) in GPMV buffer) at room temperature for 20 hr. After the incubation, GPMVs that had detached from the cells were gently decanted into a conical tube. We put then the conical tube in a 4 °C refrigerator to allow the GPMVs to settle down at the bottom of the tube for further use without any purification treatment.

### Deposition of GPMV and the air-water interface treatment

A glass coverslip was cleaned using argon plasma for 10 min. A poly(dimethylsiloxane) (PDMS)-made well was rapidly sealed using the cleaned glass to generate a cylindrically shaped space with a diameter of 1.8 cm and a height of 0.25 cm to hold solution above the glass coverslip. 80 μl GPMV solution was loaded into the PDMS well. For the GPMV deposition without any air-water interface treatment, a glass coverslip was used to cover the top of the PDMS well to reduce the water evaporation from the sample, and the GPMV solution was washed away with GPMV buffer after 1.5 hr incubation in the well.

For the air-water interface treatment, the PDMS well top was open to the atmosphere at room temperature. An air-water interface contacting the bottom glass support started to appear when the lowest point of the water meniscus reached the middle floor of the well. The rupture of the water meniscus can be clearly identified by the naked eye after around 0.5 hr in our PDMS well. After the water meniscus ruptured, the air-water interface receded to the well boundary and the entire sample looked dry after another 1 hr. For a typical sample, 80 μl of water was added to the well to rehydrate the sample after the 1.5 hr evaporation. For an over-dried sample, 80 μl of water was added 3 hr after the evaporation started (the sample stayed in the dried condition for an extra 1.5 hr). Both samples were washed with GPMV buffer before the observation.

### Immunostaining of Aquaporin 3 in HeLa cells

The sample was blocked in 2 mg/mL BSA for 1 hr at room temperature before the immunostaining. The Aquaporin 3 antibody (PE/ATTO 594, Abnova, USA) was diluted in the GPMV buffer and added into the well and incubated with the sample at room temperature at a concentration of 3 μg/mL. After 1 hr incubation, the well was extensively washed with GPMV buffer to remove the unbound antibody for further microscopic observation.

### Fluorescence recovery after photobleaching for measuring the diffusivity of Fast-DiO and Anti-AQP3 in the supported GPMV patch

We used fluorescence recovery after photobleaching (FRAP) to measure the diffusivity of Fast-DiO and Anti-AQP3 in the supported GPMV patch. A 200 mW DPSS Blue Laser Modul (SEO, Taiwan) at 473 nm and Green Laser Module (Unice, Taiwan) at 532 nm were used to bleach a small spot in membranes with Fast-DiO and Anti-AQP3 for 0.2 sec. The intensity of a bleached spot has a Gaussian profile with an approximate half-maximum width of 10 μm. Recovery images were captured using an inverted microscope (Olympus IX81, Olympus, Japan) equipped with a CCD camera (ORCA-R2, Hamamatsu, Japan). The intensity recovery time in the region of interest was processed using MATLAB (Mathworks Natick, MA, USA) to calculate the two-dimensional diffusion coefficients of the SLB. The algorithm used for this was reported in our previous study^42^ and Supplementary Information.

### Image processing to obtain GPMV patch coverage ratio

ImageJ software (NIH, Maryland, USA) was used to obtain the membrane coverage ratio. The gray scale images were converted to black and white images by using the threshold intensity determined by the methods built in ImageJ (see Supplementary Fig. S2), with the white region as the region covered by the GPMVs and the black region as the empty region with no GPMVs. The GPMV coverage ratio was further determined by the “Analyze Particle” function in ImageJ.

## Conclusions

We demonstrated the possibility of using an air-water interface treatment to efficiently break GPMVs to form supported plasma membrane patches with mobile native plasma membrane proteins. The GPMV patch coverage in the membrane platform achieved by the air-water interface treatment were seventeen times higher than those achieved by the spontaneous rupture of GPMVs. In addition, the coverage can be further significantly increased by applying multiple air-water interface treatments. The sample consisting of spontaneously ruptured GPMVs and the sample consisting of GPMVs ruptured by an air-water interface were shown to have similar averaged fluorescence intensities and membrane diffusivities, suggesting that the integrity of the GPMV patch remained after our air-water interface treatment. These supported membrane patches with native membrane proteins have planar geometry and fixed location. Incorporating the supported membranes with some surface analytical tools such as atomic force microscope (AFM) and surface plasmon resonance (SPR) could allow us to explore the membrane protein properties and ligand binding behaviors in their native-like environment. Being able to rupture tens of micron-sized GPMVs also provides the possibility for the supported membrane patches to form on porous supports for studying the transport behaviors of interested species across the plasma membrane.

## Electronic supplementary material

Supplementary Information

Supplementary Video
