# Supplementary material for: Rupturing Giant Plasma Membrane Vesicles to Form Micron-sized Supported Cell Plasma Membranes with Native Transmembrane Proteins
Source: Sci Rep. 2017 Nov 9;7:15139. doi: 10.1038/s41598-017-15103-3 (PMC5680215; doi:10.1038/s41598-017-15103-3)
Supplement: Supplementary file 1 — Supplementary Information [file 41598_2017_15103_MOESM1_ESM.pdf]

Rupturing Giant Plasma Membrane Vesicles to Form Micron-sized Supported Cell Plasma Membranes with Native Transmembrane Proteins

Po-Chieh Chiang, Kevin Tanady, Ling-Ting Huang, and Ling Chao*

Department of Chemical Engineering, National Taiwan University, Taipei, Taiwan

Email: lingchao@ntu.edu.tw

Supplementary Information

Diffusion coefficients obtained by fluorescence recovery after photobleaching (FRAP).

We used fluorescence recovery after photobleaching (FRAP) technique to examine the lipid membrane mobility. The FRAP technique uses laser to bleach fluorescent lipid molecules in a small region of the sample and the diffusion coefficients can be obtained by analyzing the fluorescence recovery with time in this small region.[1-3] The recovery time evolution images of the photobleached spot were recorded by image processing program HCImage (Hamamatsu, Japan). The intensity recovery with time inside the region of interest was then processed and fitted by MATLAB (Mathworks Natick, MA) in order to obtain the diffusion coefficient of lipids and membrane proteins in supported lipid membranes. The fitting algorithm we used for the two dimensional lateral diffusion coefficient was mainly based on the one developed by Axelrod et al.[1] and the details are in the following paragraphs.

The measured fluorescence intensity was normalized by the following equation to subtract the fluorescence background and to make it fit to the modeling equation more easily.

$$\mathrm{f_e(t)} = \frac{\mathrm{F_e(t)} - \mathrm{F_e}(0^+)}{\mathrm{F_e}(0^-) - \mathrm{F_e}(0^+)}$$

where F_e (t) is the summation of the fluorescence intensity weighted by its position away from the center of bleaching spot and can be represented below.

$$\mathrm{F_e(t)} = \frac{2qP_0}{A\pi w^2}\int C(r,t)\exp(\frac{-2r^2}{w^2})2\pi r\, dr$$

where q is the fluorophore quantum efficiency, A is the beam attenuation factor, P_0 is the total laser power, r is the position from the center of bleaching spot, w is the half width of the initial bleaching profile, and $C(r,t)$ is the fluorescence intensity measured at position=r and time= t. F_e (0^+) and F_e (0^-) are obtained at the time right after and right before the lipid bilayers are photobleached.

On the other hand, the theoretical normalized intensity can be represented in the following form.

$$f_k(t) = \frac{\mathrm{F_K(t)} - \mathrm{F_K}(0^+)}{\mathrm{F_K}(0^-) - \mathrm{F_K}(0^+)} = Mf \times \frac{\sum_{n=0}^{\infty} \frac{(-K)^n}{n!\left[1 + n\left(1 + \frac{2t}{\tau_D}\right)\right]} - \frac{1 - e^{-K}}{K}}{1 - \frac{1 - e^{-K}}{K}}$$

where *Mf* is the mobile fraction of the fluorophore. $F_K(t)$ is derived from the classic mass transport model with the assumption that the initial bleached spot has can a Gaussian intensity profile and can be represented below.

$$F_K(t) = \frac{qP_0C_0}{A}\sum_{n=0}^{\infty}\frac{(-K)^n}{n!\left[1+n\left(1+\frac{2t}{\tau_D}\right)\right]}$$

where K is bleaching parameter, C_0 is the uniform fluorophore concentration before bleaching, and τ_D is the characteristic time of diffusion.

Note that the bleaching parameter, K, and the half width of the initial bleaching profile, w, can be obtained by fitting the initial bleaching profile to a Gaussian profile.

$$\mathrm{C}(\mathrm{r}, \mathrm{t} = 0^+) = C_0\exp[-Kexp\left(^{-2r^2}/_{w^2}\right)]$$

where $C(r, t=0^+)$ is the fluorescence intensity profile at position=r and the time right after the bleaching, C_0 is the uniform fluorescence intensity right before the bleaching.

By fitting the experimentally measured $f_e(t)$ to the theoretically obtained $f_k(t)$, we can obtain two fitted parameters: the characteristic time of diffusion (τ_D) and the mobile fraction (Mf). The diffusion coefficient D can be calculated from τ_D using the following equation.

$$\mathrm{D} = \frac{w^2}{4\tau_D}$$

Deformation of giant plasma membrane vesicles (GPMVs) when the air-water interface passed through.

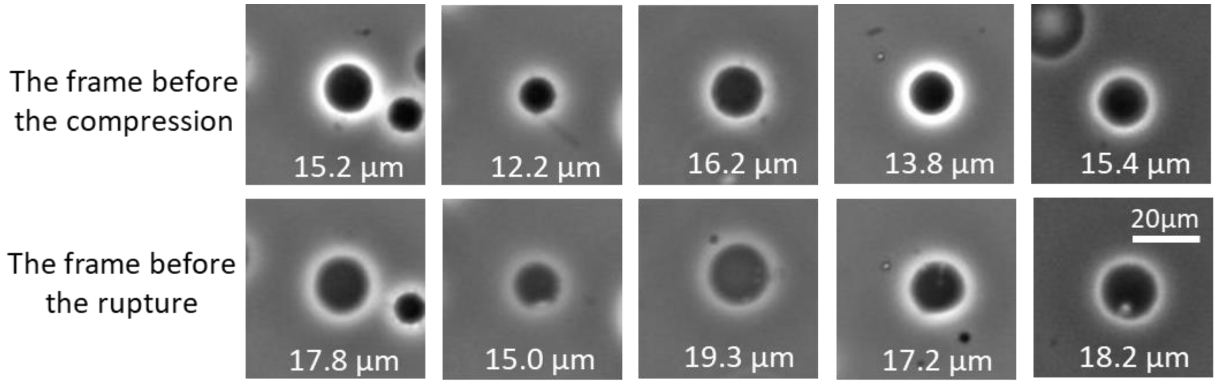

Figure S1. The image frame right before the GPMV started to be compressed and the image frame right before the GPMV ruptured to become a supported patch.

Table S1. The deformation extent of the GPMV when the air-water interface passed through. The deformation extent is the ratio of the projected area diameter right before the GPMV ruptured to become a supported patch to the projected area diameter before the GPMV started to be compressed.

Projected area diameter before compression (μm)	Projected area diameter before the rupture (μm)	Deformation extent
15.2	17.8	1.17
12.2	15.0	1.23
16.2	19.3	1.19
13.8	17.2	1.25
15.4	18.2	1.18
Average of deformation extents (n=5)		1.21±0.03
Previous study --Projected area diameter before compression estimated from the ruptured patch area (μm)	Previous study --Projected area diameter before the rupture process started (μm)	Previous study --Deformation extent
4.5	5.5	1.22

Membrane coverage data and the converted black and white images for calculating coverage ratios.

The gray scale images were converted to black and white images by using the threshold intensity determined by the methods built in ImageJ without any pre-treatment of the images. "Triangle method" in the ImageJ was chosen to convert the gray scale images of control experiments to black and white images. "Minerror method" was chosen to convert images of the samples treated with the air-water interface treatment once. "Percentile method" was chosen to convert images of the samples treated with the air-water interface treatment twice and three times. The comparisons between the raw gray scale images and the converted black and white images in Figure S2 show that the chosen methods can accurately convert the region with microscopically observable GPMV patches to the dark region and the region without microscopically observable GPMV patches to the white region. The black and white images were later used to calculate the GPMV coverage ratio by using the "Analyze Particle" function in ImageJ without any other treatment.

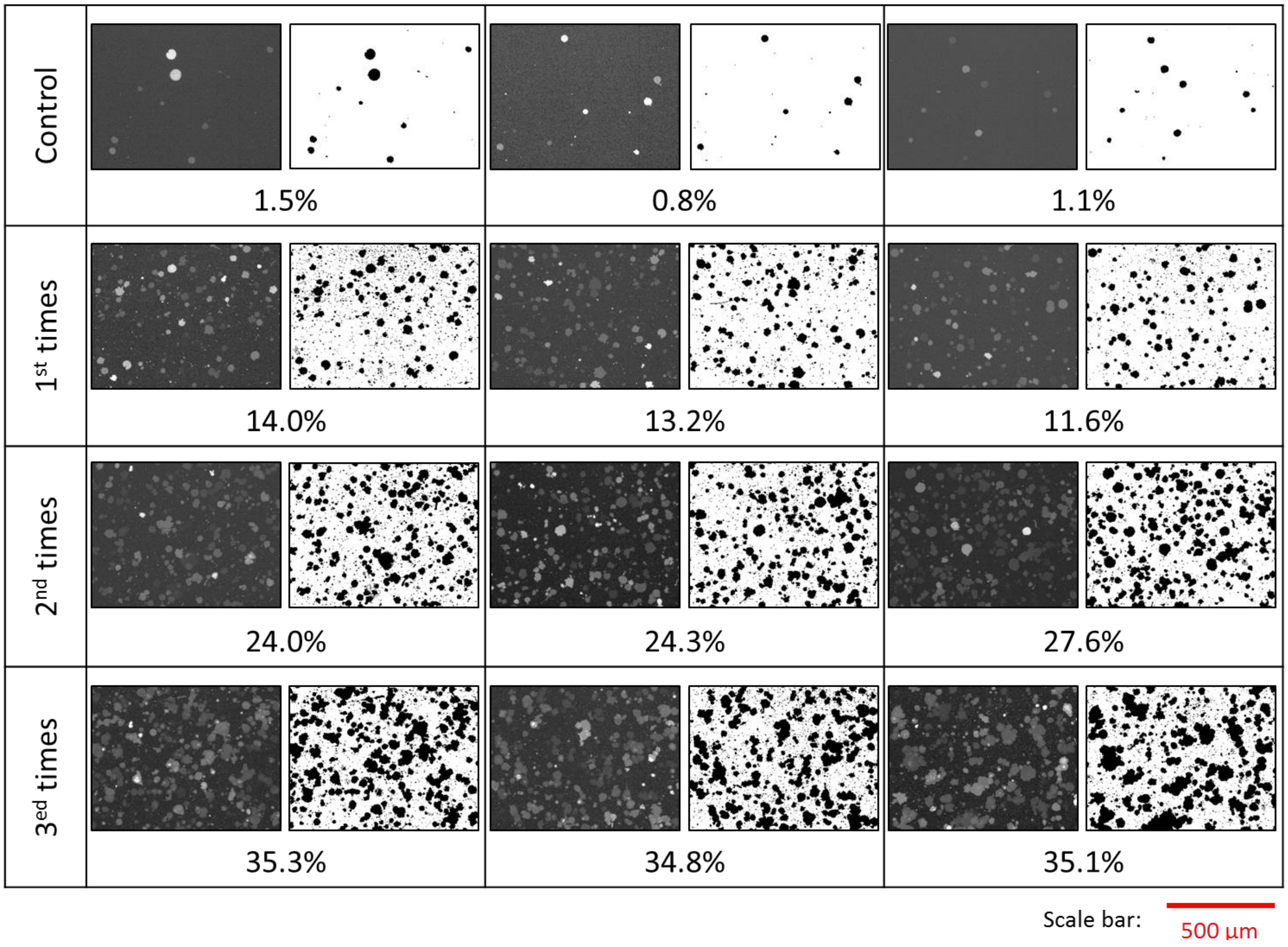

Figure S2. Membrane coverage gray scale image data and the converted black and white images for calculating coverage ratios.

Binding of Anti-Aquaporin 3 to pure DOPC membranes and GPMV patches.

We prepared two types of samples to examine the binding of anti-aquaporin 3(anti-AQP3). The first one is the control supported lipid bilayer composed of pure DOPC (Figure S3(a)). The second one is the GPMV patches on the glass blocked with 2 mg/mL BSA for 1 hr at room temperature before the immunostaining (Figure S3(b)). 3 μg/mL Aquaporin 3 antibody (PE/ATTO 594, Abnova, USA) was incubated with the samples at room temperature for 1 hr and washed away for the microscopic observation. The fluorescence intensity (FL) of Figure S3(a) is much lower than those in Figure S3(b), suggesting that the nonspecific binding of the antibody to a typical supported lipid membrane is weak.

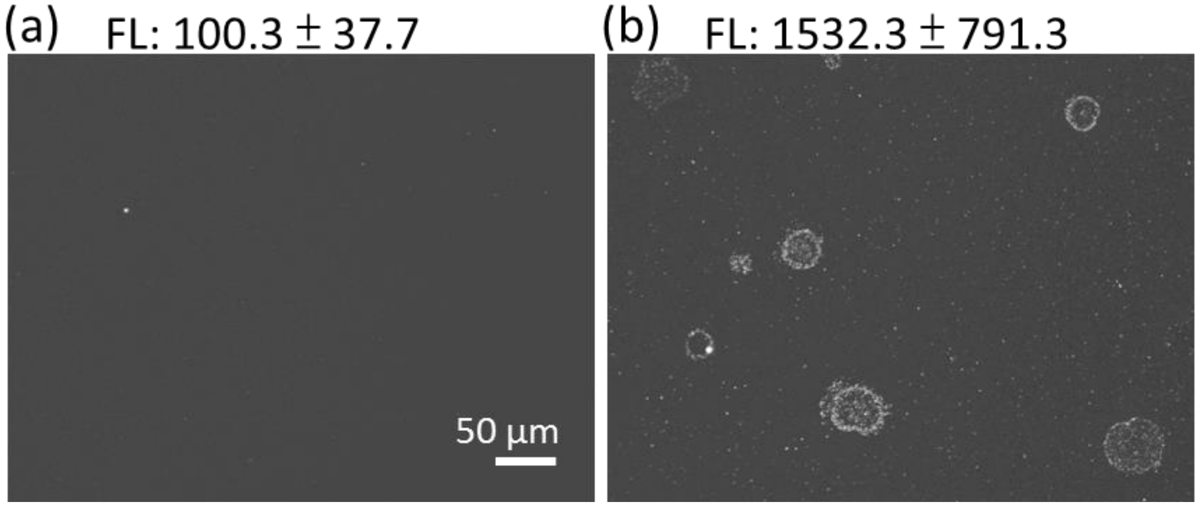

Figure S3. Fluorescence images of anti-AQP3 (a) in the pure DOPC supported lipid membrane; (b) in the sample with GPMV patches with the surrounding region blocked by BSA. FL: the averaged fluorescence intensity of the entire image.

Fluorescence Recovery of the Anti-AQP3 bound to the Boundaries of the GPMV Patches.

We noticed that anti-AQP3 preferred to bind to the boundaries of the GPMV patches. We photobleached the fluorescently labeled antibodies at the patch boundaries and found that the fluorescence can recover (Figure S4), indicating that these bright antibody rims were not due the preferential non-specific binding of the antibodies to the patch boundaries. We hypothesized that membrane proteins like AQP3 may have flexibility to deform to protect the exposed hydrophobic acyl chains of the bilayer at the patch boundaries and therefore were trapped at the membrane patch boundaries to decrease the system energy.

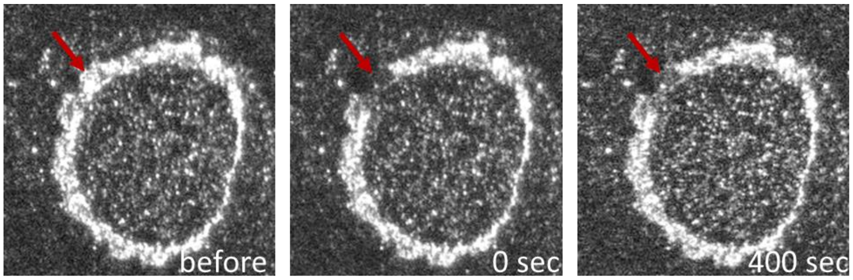

Figure S4. Fluorescence recovery of anti-AQP3 after photobleaching at the GPMV patch boundaries. The surrounding region of the GPMV patch was blocked by BSA. Red arrows indicate where the photobleaching was performed. The left image were taken before the sample was photobleached. The middle and right images were taken right after the photobleaching and 400 seconds after the photobleaching.

References

1. D. Axelrod, D. E. Koppel, J. Schlessinger, E. Elson and W. W. Webb, *Biophys J*, 1976, **16**, 1055-1069.
2. T. V. Ratto and M. L. Longo, *Biophysical Journal*, 2002, **83**, 3380-3392.
3. M. Kang, C. A. Day, K. Drake, A. K. Kenworthy and E. DiBenedetto, *Biophysical Journal*, 2009, **97**, 1501-1511.
